# The Power of Touch: Type 4 Pili, the von Willebrand A Domain, and Surface Sensing by Pseudomonas aeruginosa

**DOI:** 10.1128/jb.00084-22

**Published:** 2022-05-25

**Authors:** Shanice S. Webster, Gerard C. L. Wong, George A. O’Toole

**Affiliations:** a Department of Microbiology and Immunology, Geisel School of Medicine at Dartmouth, Hanover, New Hampshire, USA; b Department of Bioengineering, University of California, Los Angeles, Los Angeles, California, USA; c Department of Chemistry and Biochemistry, University of California, Los Angeles, Los Angeles, California, USA; d California NanoSystems Institute, University of California, Los Angeles, Los Angeles, California, USA; University of Southern California

**Keywords:** type 4 pili, force, PilY1, von Willebrand A domain, surface sensing

## Abstract

Most microbes in the biosphere are attached to surfaces, where they experience mechanical forces due to hydrodynamic flow and cell-to-substratum interactions. These forces likely serve as mechanical cues that influence bacterial physiology and eventually drive environmental adaptation and fitness. Mechanosensors are cellular components capable of sensing a mechanical input and serve as part of a larger system for sensing and transducing mechanical signals. Two cellular components in bacteria that have emerged as candidate mechanosensors are the type IV pili (TFP) and the flagellum. Current models posit that bacteria transmit and convert TFP- and/or flagellum-dependent mechanical force inputs into biochemical signals, including cAMP and c-di-GMP, to drive surface adaptation. Here, we discuss the impact of force-induced changes on the structure and function of two eukaryotic proteins, titin and the human von Willebrand factor (vWF), and these proteins’ relevance to bacteria. Given the wealth of understanding about these eukaryotic mechanosensors, we can use them as a framework to understand the effect of force on Pseudomonas aeruginosa during the early stages of biofilm formation, with a particular emphasis on TFP and the documented surface-sensing mechanosensors PilY1 and FimH. We also discuss the importance of disulfide bonds in mediating force-induced conformational changes, which may modulate mechanosensing and downstream biochemical signaling. We conclude by sharing our perspective on the state of the field and what we deem exciting frontiers in studying bacterial mechanosensing to better understand the mechanisms whereby bacteria transition from a planktonic to a biofilm lifestyle.

## INTRODUCTION

Touch has a memory.

—John Keats

The effects of mechanical forces on eukaryotic systems were recognized more than 100 years ago (1) and are a driving factor for embryogenesis, morphology, development, and disease (2–4). Macroscopic organisms and tissues encounter not only intricate chemical environments but also complex and dynamic mechanical signals that they must sense and eventually convert into cellular responses in order to survive and proliferate (5, 6). It is no different for bacteria and archaea. Indeed, the idea that bacteria experience and respond to physical forces has been in the literature for decades (7, 8). Koch et al. (7) theorized that bacterial growth and morphology are due to internal hydrostatic pressures acting on regions of the peptidoglycan (9). Advances in microscopy and microfluidics later confirmed that compressive forces affect turgor pressure and influence bacterial cellular geometry (10). In natural and clinical environments, bacteria experience a variety of physical forces (11). On living surface such as plant roots or the lungs of persons with cystic fibrosis or on non-living surfaces, such as rocks or soil particles, physical perturbations occur as shear and adhesive forces. Shear forces are defined as unaligned forces that act on one part of a cell in one direction and another part of the same cell in the opposite direction while adhesive forces are those associated with cell-to-substratum contact.

While there is ample evidence to indicate that microbes can sense the presence of a surface, how this type of sensory input is transduced into signals that elicit a cellular response is much less clear. The purpose of this review is to (i) summarize concepts relevant to the microbial sense of touch, including what is known historically about eukaryotic force-sensing proteins, and (ii) critically engage representative sensing models proposed in the current literature and highlight where open questions remain. We focus on how the type IV pili (TFP), the TFP tip-associated protein PilY1, and the flagellum drive surface sensing by Pseudomonas aeruginosa. Our hope is that the findings and questions summarized here can provide an informing context to guide future work.

## A NOTE ON NOMENCLATRUE

Bacterial mechanobiology is a new field that has borrowed many of the terms used to describe the impacts of force from eukaryotic literature; it is therefore important to clearly define the terms adopted in the bacterial literature to ensure consistency. In eukaryotes, mechanosensing is an overarching term used to describe the ability to sense a mechanical force from the environment, including but not limited to flow and surface contact (12, 13). A mechanosensor is any cellular component or protein that undergoes a state change, such as unfolding, or a conformation change, in response to a mechanical stimulus (14). In eukaryotic mechanobiology, mechanical stimuli are often directly sensed by mechanosensitive proteins present at the cell membrane, such as integrins, growth factor receptors, and stretch-activated ion channels (15), or are directly sensed by cell components/proteins suspended in liquid, such as platelets sensing the stiffness of fibrinogen in the blood plasma (16). In bacteria and archaea, known mechanosensing proteins were initially limited to the mechanosensitive channels (MSCs) in the plasma membrane that directly sense turgor pressure (17), but in the recent bacterial literature, the term mechanosensor has also been applied broadly to proteins that are likely not proximal to the mechanical stimuli. In our opinion, echoed in the recent excellent review by Chawla et al. (18), the loose application of the term in this “indirect” sense has resulted in some confusion. In particular, that the meaning of the term has become progressively more general (especially in the context of bacterial mechanosensing) has made conceptual linking of past and present literature more difficult than it needs to be. Although mechanosensing and surface sensing are often used interchangeably, these terms have distinct meanings. Surface sensing, the more general term, refers to the mechanical and chemical cues that allow the perception of the proximal presence of a surface (19). For example, for Pseudomonas fluorescens, inorganic phosphate promotes biofilm formation; because this nutrient is liberated from iron-phosphate minerals, this surface-bound nutrient is hypothesized to serve as a surrogate signal for a surface (20), a case of chemically cued surface sensing. Similarly, in Pseudomonas aeruginosa, the exopolysaccharide Psl acts as a signal to stimulate the diguanylate cyclases SadC and SiaD to increase cyclic di-GMP production and create a positive-feedback loop that promotes biofilm formation (21). Mechanosensing specifically refers to mechanical force and therefore applies to only a subset of surface-sensing mechanisms; a response to mechanical force should be formally demonstrated if this term is used. Finally, in the “eukaryotic world,” the transmission of an external mechanical signal into the cell is referred to as mechanotransmission, and the subsequent conversion of that signal into a biochemical response is termed mechanotransduction (13). For simplicity and in accordance with the terminology used in bacterial biology, we use the term “signal transduction” to include the signal transmitted into the cell and the ultimate cellular response that is elicited, independent of whether the signal is chemical or physical. That is, we argue that there is nothing “magical” about mechanical signals; they are inputs like any other and more than likely use standard mechanisms of bacterial signal transduction. In fact, in the case of bacteria, some of these signals piggyback on chemosensory signal transduction networks (19).

## DISULFIDE BONDS AS SWITCHES FOR PROTEIN FUNCTION

**FIG 1:**
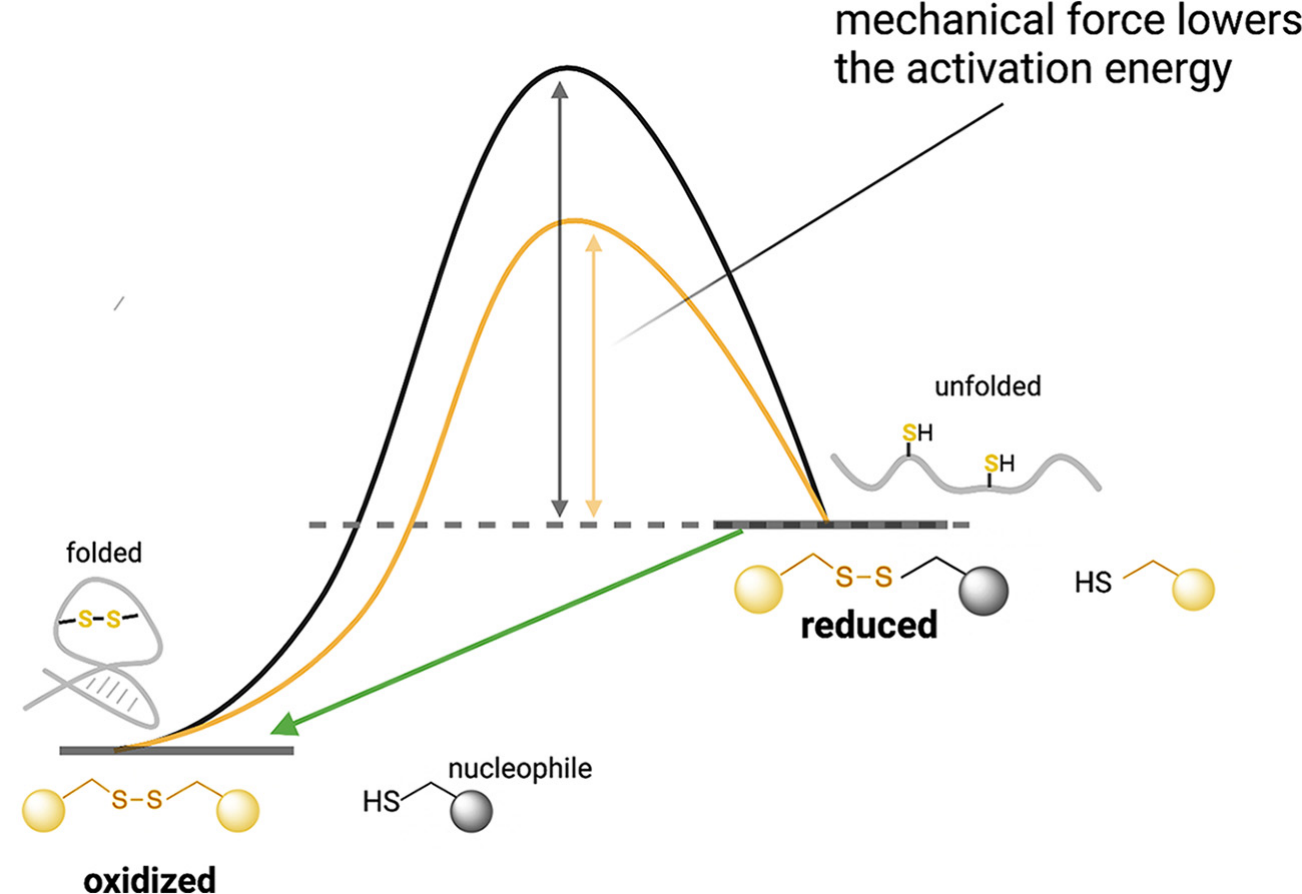
Energy diagram showing the effect of mechanical force on the redox state of sulfhydryl groups and disulfide bonds and the consequences for protein unfolding/folding. Mechanical force lowers the activation energy (yellow curve) required to break protein disulfide bonds, a thermodynamically unfavorable reaction (Δ*G* > 0). Sufficient force puts the protein in a high-energy strained conformation, making it susceptible to nucleophilic attack. Due to the high-energy state of the products compared to the reactants, the reaction can be spontaneously reversible (green arrow), and the protein can revert to its original folded state (23). Note, however, that there are also cases where the application of force stabilizes a disulfide bond, effectively increasing the activation energy (32).

In typical chemical reactions, participating molecules need to overcome an activation energy barrier before they can react to form products. This required energy is usually provided as thermal, electrical potential, or light energy. However, mechanical forces can also lower the activation energy and allow thermodynamically unfavorable reactions, such as protein conformation changes, to become favorable. For example, they can lower the energy barrier by disrupting hydrogen bonds or van der Waal interactions within a protein and so cause the protein to transition from a folded to a partially unfolded state. These changes in protein conformation may result in new biological or biochemical properties or downstream effects (22, 23) (Fig. 1). More generally, mechanical forces can deform reactant molecules along a specific direction of the reaction coordinate and thereby bias specific reaction pathways that make products unattainable through other reactions (24).

Many native proteins that function in the extracellular environment and encounter mechanical forces *in vivo*, like titin and von Willebrand factor (vWF), have disulfide bonds (25). Initially, it was thought that the formation of disulfide bonds is largely irreversible and that their sole functions are to maintain protein structural integrity and confer protein rigidity (26, 27). However, they not only are redox active (Fig. 1) but also can act as switches for a myriad of protein functions (28, 29). The redox potential of disulfide bonds can be influenced by mechanical forces that occur physiologically (i.e., in the context of native biological systems) and that induce protein conformational changes (30), which in turn can impact protein function.

Breaking disulfide bonds is an energetically unfavorable process. However, with sufficient mechanical force applied, the protein attains a high-energy strained conformation that facilitates the breaking of the disulfide bond. The return to the protein’s original conformation once the force is removed can be spontaneous (23) but not in all cases. For example, specific conditions, such as an oxidizing environment, are required for the reverse reaction. Furthermore, if there are multiple disulfide bonds, then there is the potential for cooperative effects (27) or off-pathway disulfide bond formation. Disulfide bonds can play critical roles in holding an intrinsically frustrated protein (e.g., a protein folded into a structure with high residual internal stress, which thereby has an increased propensity to unfold) together, which implies that the disulfide bonds’ context-dependent interactions with external forces may exhibit especially interesting phenomena (31). While mechanical force has the potential to break disulfide bonds, in some circumstances, such forces can make disulfide bonds stronger, rather than weaker, by changing the bond’s conformation to be more resistant to nucleophilic attack (32). The modulation of the redox state of a disulfide bond by mechanical forces indicates that external mechanical stress has the potential to be transduced into a biochemical signal to regulate biological phenomena, including but not limited to mechanosensing.

## ROLE OF SHEAR STRESS AND DISULFIDE BONDS IN REGULATING THE FUNCTION OF EUKARYOTIC MECHANOSENSORS

### Function of the vWF.

**FIG 2:**
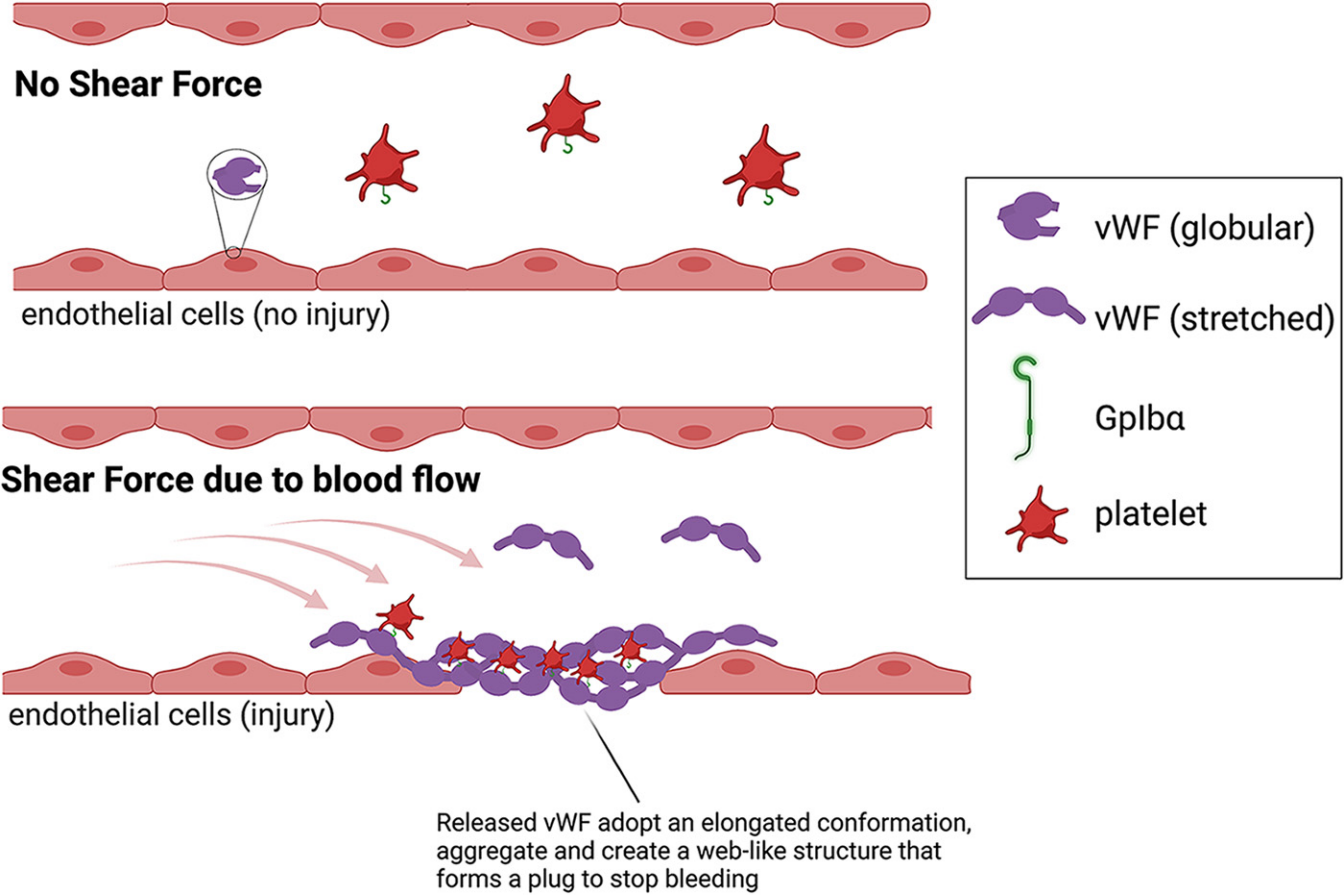
Function of the von Willebrand Factor (vWF) in blood clotting. (Top) In the absence of vascular damage, where there is low shear force, the vWF protein present in endothelial cells adopts a globular conformation. (Bottom) During vascular damage, vWF is released from endothelial cells (53), and increased shear forces due to blood flow result in the protein adopting a stretched conformation (48, 54, 55) that binds collagen, aggregates to form a web-like structure at the site of injury, and binds to GpIbα on platelets.

The vWF is a large multidomain adhesive glycoprotein that is important to stem bleeding and is a mechanosensor of shear force (33, 34). A deficiency or defect in vWF leads to a number of blood and bleeding disorders, the most common being the inherited von Willebrand disease (35, 36). When vascular damage occurs, vWF is released from endothelial cells (Fig. 2). Next, shear forces due to blood flow cause vWF to undergo conformational changes that modify its structure from a globular to an elongated or stretched conformation. The stretched conformation of vWF allows this protein to both adhere to collagen and bind to the GpIbα protein found on platelets, thereby mediating the adhesion of platelets to sites of vascular damage (37). Higher shear forces can also cause vWF to self-associate, forming aggregates that create a spiderweb-like structure that traps platelets at the site of vascular damage to form a plug and stop bleeding (37). In addition to binding platelets, the stretched conformation of vWF is subjected to proteolysis by the zinc protease ADAMTS-13 (a disintegrin and metalloproteinase with a thrombospondin type 1 motif, member 13), which fragments vWF and limits its prothrombic activity, thereby maintaining equilibrium between hemostasis (blood clotting) and thrombosis (excessive blood clotting) (38).

### The A domains, platelet binding, and intramolecular disulfide bonds in vWF function.

**FIG 3:**
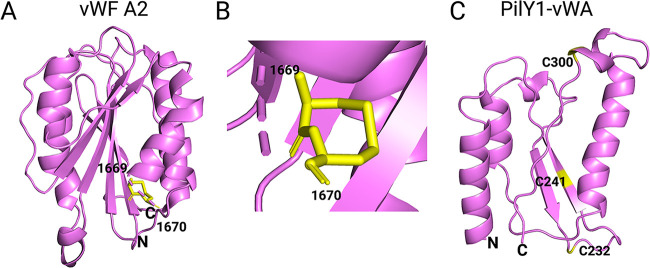
Structures of the vWF-A2 domain and the vWA domain of PilY1. (A) Ribbon diagram of the structure of the VWF A2 domain (PDB accession number 3GXB) (40) showing the C-terminal vicinal disulfide bond between Cys1669 and Cys1670 in yellow. The domain has the classic Rossman fold, i.e., central β-sheets surrounded by α-helices. The N and C termini are labeled. (B) The eight-membered vicinal disulfide ring (40) shown at a higher magnification. (C) Ribbon diagram showing the predicted structure of the vWA domain of PilY1 generated using Phyre (159). The domain also shows a classical Rossmann fold (151) consisting of central β-sheets surrounded by α-helices. This predicted structure was generated using homology to the vWF A2 domain (PDB accession number 3GXB) (40); amino acids 201 to 203, 206 to 230, 234 to 272, 273 to 286, and 299 to 317 of the vWA of P. aeruginosa PA14 are mapped. Three of the seven cysteines (C232, C241, and C300) are shown in yellow, and the N and C termini are labeled.

Disulfide bonds are formed between identical vWF domains, resulting in the formation of a multiprotein complex as large as 20,000 kDa. The vWF is comprised of the A domain, which consists of the repeats A1, A2, and A3, plus the C and D domains. The A1 and A2 domains are located next to each other and are connected by a 30-amino-acid linker (39). Each A domain contains a pair of cysteines, but the A2 domain is quite distinct from the A1 and A3 domains in that its pair of cysteines is linked by adjacent disulfide bonds at the C terminus via a vicinal disulfide bond, Cys1669-Cys1670. The result is an eight-member ring that provides rigidity to the A2 domain (40, 41) (Fig. 3A and B). This disulfide ring is proposed to act as an energy barrier that senses forces acting on the C terminus of the A2 domain; if this energy barrier is overcome by shear forces, the force-derived signal is thought to propagate to the hydrophobic core of the A2 domain, leading to unfolding (40, 42). Aponte-Santamaría and colleagues demonstrate that under low shear force or equilibrium conditions, the A1 domain interacts with the A2 domain and occludes the platelet-expressed protein GpIbα from binding to the A1 domain of vWF (43). Through force probe molecular dynamics simulations in combination with atomic force microscopy (AFM), wherein a stretching force is applied to the A1-A2 complex, it was observed that the A2 domain unfolds, and the shielding effect of A2 is eliminated, exposing the A1 domain to GpIbα binding. In these experiments, it is unclear whether the stretching force cleaves the vicinal disulfide (Cys1669-Cys1670) in the A2 domain. In a separate study, Butera and colleagues demonstrate that only when the Cys1669-Cys1670 bond is cleaved and force is then applied is A2 binding to A1 relieved, and GpIbα binding is promoted (44). They argue that when the vicinal disulfide is intact, autoinhibition of GpIbα-A1 binding by the A2 domain remains intact, independent of the force applied. Thus, the exact role of the vicinal disulfide in mechanosensing remains to be resolved. The current model is that hydrodynamic forces allow the transition of vWF from a globular to a stretched conformation, and vWF is then competent for GpIbα binding in the stretched conformation.

### Ideal, catch, and slip bonds in the function of the vWF mechanosensor.

At sites of vascular injury, the platelet surface protein GpIbα binds to the vWF A1 domain under high shear forces (37). Maintaining the balance of the vWF-GpIbα interaction is key to ensuring sufficient adhesion to the site of injury to stop bleeding but not so much that thrombosis occurs (45). This delicate balance is mediated by the bonds that form between the vWF A1 domain and the GpIbα protein. To describe interactions in mechanosensing, it is helpful to define a taxonomy of “dynamic bonds.” In a seminal 1988 paper, Dembo et al. showed that it is possible for bond dissociation to be affected by force in fundamentally different ways (46). Bonds that are strengthened by force, weakened by force, and independent of force are defined as “catch bonds,” “slip bonds,” and “ideal bonds,” respectively. A catch bond is a dynamic, noncovalent interaction that displays a counterintuitive behavior in which both bond strength and bond lifetime increase with applied tensile force until some optimal magnitude of force is reached, above which the catch bond will weaken with increased force (46, 47). This behavior is in contrast to those of slip bonds, which are noncovalent interactions where force decreases the bond strength and bond lifetime. For ideal bonds, bond strength and lifetime are independent of applied force. vWF participates in all three types of bonds during its function in blood clotting (48).

vWF binds to collagen at two sites, its A3 domain, which specifically binds type I and III collagens (49, 50), and its A1 domain, which binds primarily to collagen VI (51). The interaction between vWF multimers and collagen is regulated by ideal bonds since binding is not affected by the magnitude of shear forces. vWF multimers bind to collagen at a wide range of forces varying from low physiological to high shear rates of 400, 1,700, and up to 4,000 s^−1^ (52). Other studies demonstrate that regardless of the conformation, stretched or globular, force or no force, vWF still binds to collagen, highlighting the formation of ideal bonds (53).

Several studies show that the interaction between GpIbα and vWF, specifically vWF’s A1 domain, is governed by a catch-slip bond regime (48, 54, 55). The Zhu group coated microspheres with GpIbα that was transiently tethered to the vWF A1 domain. Those researchers then subjected microspheres-vWF-GpIbα to increasing forces in a flow chamber and measured the bond lifetimes. Those authors observed a characteristic biphasic pattern of an initial increase in the bond strength up to an optimal force of 20 pN (catch bond) and a subsequent decrease in the bond strength as the force increased to 120 pN (slip bond) (48). Molecular dynamic simulations (55) also show a similar biphasic pattern. Consistent with these observations, the Zhu group also performed flow chamber studies with the vWF-R1306Q variant, with a mutation in the A1 domain that occurs in some patients with type 2 von Willebrand disease (56), and found that the bond lifetime decreases with increased force, a behavior characteristic of slip bonds. In this mutant, the catch bonds that were observed with wild-type (WT) vWF A1 were converted to slip bonds (48). Interestingly, the vWF A1-GpIbα interaction was initially shown to flex between two slip bond states (57); however, the discrepancy between the studies was later found to be due to different recombinant sequences used for the vWF A1 domain (54). Overall, these data indicate that a single mutation can alter fundamental aspects of vWF’s mechanical behavior.

### Force and disulfide bonds as drivers of protein unfolding and the elasticity of titin.

**FIG 4:**
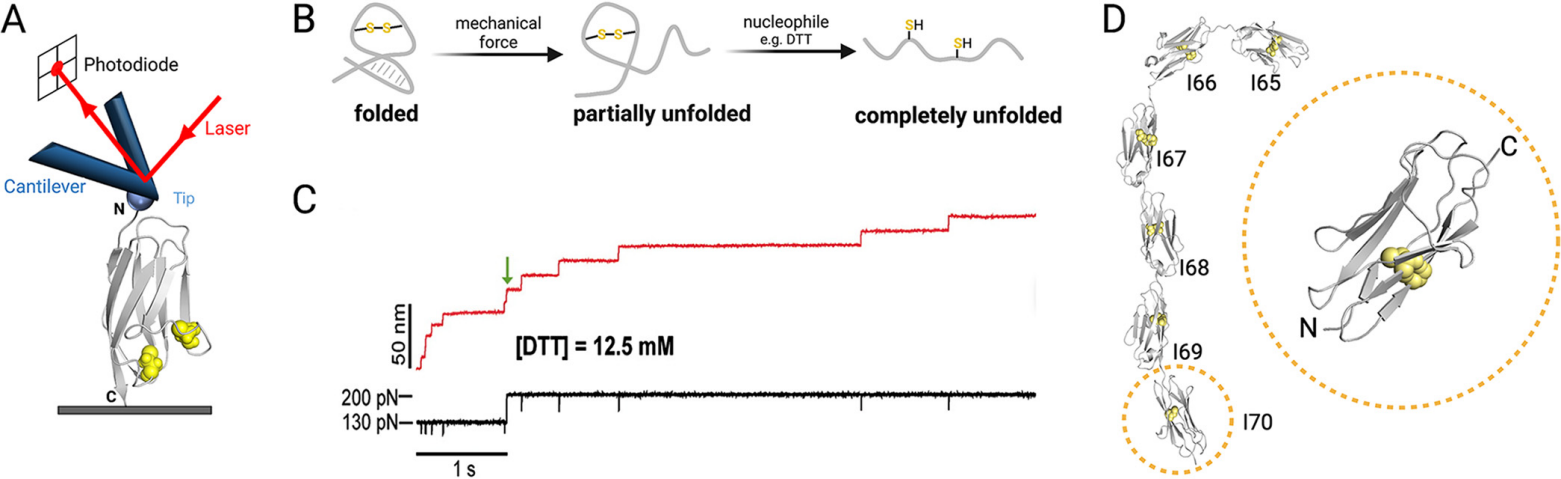
Mechanical force can induce protein unfolding shown in AFM studies with titin. (A) Schematic diagram of a force spectroscopy experiment where a functionalized AFM tip is used to probe the I27 domain of human cardiac muscle titin (PDB accession number 1TIT) (160). Cysteines in the protein are highlighted as yellow spheres, and the N and C termini are labeled. The protein is probed with a constant force or at a constant speed. When the tip encounters the protein, the tip starts to retract, pulling on the protein and exerting a force that causes the protein to unfold until it reaches a maximum (rupture) length. (B) Transition of a protein from a folded to a completely unfolded state. Mechanical force breaks hydrogen bonds and other intramolecular interactions within the protein, leaving the region enclosed by the disulfide bond still folded. In the presence of a reducing agent and force (or very high force alone), the protein completely unfolds, and the residues occluded by the original disulfide-bond-stabilized structure are fully exposed. (C) Double-pulse force clamp experiment for the I27 domain of titin with an engineered disulfide bond (22). An initial pulse of 130 pN causes an ~11-nm increase in the length of the protein, which corresponds to the partial unfolding of the protein. In the presence of dithiothreitol (DTT) (green arrow), and when the force is increased to 200 pN, steps of ~14 nm are observed, reflecting the unfolding of the region enclosed by the disulfide bond. (Republished from reference 22 with permission of the publisher.) (D) Crystal structure of the rabbit titin segment I65–I70 (PDB accession number 3B43) (161) marked by six tandem Ig domains. The protein contains 14 cysteines (yellow spheres) distributed among six Ig domains. The inset shows a magnified view of the I70 domain.

Titin is one of the best-studied mechanosensory proteins. It is a large protein that functions as a molecular spring and is essential for muscle contraction and maintaining the elasticity of the heart. Titin can reversibly stretch and then refold into a more compact state during cycles of heart resting and contraction, respectively (58). The titin filament consists of numerous immunoglobin (Ig)-like domains, including the Ig-like domain I27 (Fig. 4A), which mechanically unfolds and refolds (58). Single-molecule force clamp spectroscopy studies using the I27 domain of cardiac titin, which consist of two engineered cysteines that form a single disulfide bond, show that force triggers disulfide bond reduction (23, 59). Using a double-pulse protocol that first applies a constant force to the 8 repeat units of the I27 polyprotein, the Fernandez and Garcia-Manyes groups showed a defined stepwise increase in the length of the polyprotein that marks the unfolding and extension of its eight individual domains (22, 23, 59, 60) (Fig. 4B and C). Specifically, the protein increases in length in steps of ~11 to 15 nm when pulled at a constant force of ~130 pN due to the breaking of hydrogen bonds that trigger unfolding. After this first round of unfolding, the disulfide bonds are still intact (Fig. 4C). Application of the second pulse alone, wherein the force is increased to 200 pN, is not sufficient to break the disulfide bond (22, 23), perhaps because forces in the nanonewton range (1 to 2 nN) are required to break covalent bonds such as disulfides (61).

It should be noted, however, that other studies have revealed that pulling forces even as small as 100 pN, in combination with changing dihedrals and bond angles, contribute to the destabilization of disulfide bonds (30) and that forces on the order of 100 pN create tensile stress that can destroy disulfide bonds (62). In the presence of hydroxide ions and reducing agents of various strengths, which can perform nucleophilic attacks on the disulfide bond, the I27 domain shows additional extension steps due to further protein unfolding as a result of releasing the region enclosed by the disulfide bond (23) (Fig. 4B and C). While force does not directly break the disulfide bonds in these experiments, it partially unfolds the protein and makes the disulfide bonds accessible to nucleophilic attack by the reducing agent. A key finding in these studies is that upon the removal of force, the reformation of the disulfide bond is spontaneous due to the higher-energy state of the cleaved disulfide than of the intact oxidized protein (23) (Fig. 1). The role of the reversible, force-dependent cleavage of the disulfide bond is analogous to the transitions thought to occur for the vicinal disulfide in the vWF A2 domain (40, 42). However, while titin and vWF act like springs, this behavior should not be taken as the only consequence of mechanical force on proteins; we outline additional possible behaviors in our discussion of PilY1’s von Willebrand A (vWA) domain below.

The redox state of disulfide bonds has been put forward as a likely mechanism to control the elasticity of titin. Interestingly, close to half of the Ig domains of titin contain cysteines (63) at positions that would allow the formation of disulfide bonds (64). Inspired by the structure of rabbit titin, which contains 6 tandem Ig modules with 14 cysteines in the I65–I70 region (Fig. 4D), Giganti and colleagues demonstrated that the oxidation of the Ig domains leads to the formation of nonnative disulfide bonds that cause the protein to be rigid (25). Interestingly, those authors showed that this configuration allows mechanical force-induced disulfide isomerization and, eventually, disulfide cleavage. The disulfide-dependent conformational states of the protein, which are modulated by the mechanical and redox environment of the cardiac muscle, are thought to fine-tune the unfolding process. Thus, studies show that mechanical forces can affect the redox state of disulfide bonds, constituting a mechanism by which these proteins can change their conformation to perform their specific function(s).

## OUR CURRENT UNDERSTANDING: MODELS FOR SURFACE SENSING IN BACTERIAL SYSTEMS

Presumably, bacteria have mechanosensing systems that respond to force. We first discuss current models whereby bacteria can detect surface engagement. In many cases, the potential players in surface sensing have been identified. However, for these examples, the mechanism whereby surface contact is sensed and the signal is transduced and whether the proximal signal is indeed mechanical force are still open questions. Next, we describe two cases, FimH of Escherichia coli and PilY1 of P. aeruginosa, that satisfy the criteria typically used in eukaryotic systems to dub a protein a “mechanosensor.”

### Sensing membrane and cytoskeletal perturbations.

Envelope proteins of the Rcs and Cpx pathways of Proteus mirabilis and Escherichia coli, respectively, are thought to sense the deformation of the cell membrane or periplasm upon surface contact (65, 66). The activation of the Rcs pathway (67) and the loss of CpxR-regulated genes (68) disrupt biofilm formation, indicating a link between membrane perturbation and the initiation of attachment. However, a direct connection between surface-induced membrane deformation, surface engagement, and Rcs signaling has not been established.

Similarly, the MscL and MscS mechanosensitive channel proteins, which act as emergency release valves during osmotic stress, directly respond to mechanical tension on the membrane (69, 70). Recent studies indicate that these channels open in response to a loss of lipidation because of the tension applied to the membrane (71); thus, a model is emerging as to the proximal signal that triggers these channels. Triggering of the MscL channel has been linked to surface engagement by Staphylococcus aureus (72), although a general role in biofilm formation for these channels is still unclear. Furthermore, these Msc proteins have not been identified in the numerous screens looking for mutants defective in early biofilm formation by pseudomonads (73–81).

### Homologs of eukaryotic mechanosensors in bacteria.

Like the extracellular matrix and cytoskeleton of eukaryotes that help to maintain cell shape/organization and provide mechanical support, bacteria and archaea have homologous proteins such as MreB and FtsZ. MreB is an actin homolog and is critical for maintaining rod-shaped morphology in bacteria such as E. coli (82). MreB affects not only the growth rate of the cell wall but also how the growth rate scales with the cell’s geometry (10). Loss of MreB function has been associated with altered biofilm formation, likely due to impacts on cell shape (83–85). FtsZ is a homolog of the eukaryotic protein tubulin. FtsZ forms a ring at the site of cell division that generates a constriction force (86). FtsZ is regulated by the Rcs system (87), although a clear role for this essential protein in biofilm formation is also difficult to discern due to its impact on viability (88–90).

### Sensing flow: another mechanical cue?

Bacteria may also sense and respond to mechanical cues other than surface engagement in their environment; fluid flow has been proposed as one such signal. In an interesting study by Sanfilippo et al., the authors discovered an operon (*fro*) in P. aeruginosa whose expression was modulated by the fluid flow rate (91), analogous to the increased expression of the locus of enterocyte effacement virulence factors observed in E. coli in the presence of flow (92). Interestingly, Sanfilippo et al. also argued that this ability to sense flow was not dependent on sensing forces since the expression of the *fro* operon was dependent on the shear rate but not the solution viscosity. This notion challenges the overarching paradigm that bacteria sense the intensity of fluid flow via the detection of forces. Given that flow apparently regulates the c-di-GMP levels of P. aeruginosa (93), and given that fluid flow can, in principle, be sensed during bacterial swimming, it would be illuminating to determine whether fluid flow is a necessary or sufficient condition for driving *fro* gene expression.

### The flagellum in mechanosensing.

The flagellum was perhaps the first proposed mechanosensor in bacteria. Numerous studies have implicated the flagellum in surface detection and biofilm formation (94–97), and a few have shed light on its proposed role specifically in mechanosensing. A pioneering study by McCarter et al. in 1988 described the polar flagellum of Vibrio parahaemolyticus as a dynamometer (98); that is, they proposed that the polar flagellum participates in mechanosensing by detecting forces that obstruct rotation, whereupon a signal transduction pathway triggers the production of lateral flagella needed for swarming motility. In this case, signal activation was observed when the bacterium was either grown on an agar surface or suspended in a viscous medium. The McCarter group later determined that the production of lateral flagella was regulated by SrcC, a GGDEF and EAL dual-domain protein that functions as a phosphodiesterase *in vivo* to modulate lateral flagellum synthesis and swarming motility (99). However, the full signaling pathway has yet to be elucidated, and there is no direct evidence that the flagellum is the mechanosensor in this system.

In a 2017 study in *Caulobacter*, Hug et al. also propose the flagellar machinery as a mechanosensor. In their model, the stator subunits, rather than the external parts of the flagellum, act as the sensor (100). In this “tetherless model,” interference with stator function, rather than with the flagellar appendage itself, stimulates c-di-GMP production and activates holdfast synthesis upon surface contact (100). A role for stators in surface sensing has also been reported for P. aeruginosa (96, 101). Furthermore, Hug et al. propose that the sole role of TFP retraction is to position the flagellum in proximity to the surface, and they argue against a role for TFP in surface sensing (we address this idea in more detail below). While this work supports a role for the flagellar motor as a surface sensor, whether the signal transduced is mechanical in nature is still an open question; perhaps, the signal is related to stator occupancy or proton flow. Indeed, a change in pH due to proton flow was one of the models proposed by those authors. Furthermore, if the signal is indeed mechanical, how stators sense it and then transduce it into a cellular response is still unclear.

How might flagellar motors, specifically their stator components, act as mechanosensors? Flagellar motors require increased power to rotate when in a viscous environment or when the bacterium engages a surface (think of a surface-attached flagellar tail wagging the cell body); this increased power is provided by recruiting more stator subunits to the motor in a load-dependent manner (102, 103). More stators generate additional torque to maintain rotation. The ability of stators to sense load suggests that they are mechanosensitive. Intriguingly, the ability of stator subunits to directly sense load would be consistent with the recently described formation of catch bonds, whereby the lifetime of an assembled stator subunit in the motor increases at higher forces (104). This is a potential molecular strategy for bacterial cells to sense and respond to increased mechanical load on the flagellum. How, then, do findings on load-dependent stator insertion (102, 103) and the work of McCarter et al. (98) square with the work of Hug et al. (100), who postulate that the flagellar appendage is dispensable for surface sensing? They might not need to, as all three of these reports were performed with different organisms. Or perhaps stator occupancy of the motor drives signaling: might the stators fully occupy the motor under maximal load or minimal load? That is, whether the flagellum has stopped rotating because it is bound to a surface or because the flagellum has been removed by mutation may make no difference to the cell; the same signaling output may be generated. Understanding the role of the flagellum, stators, and/or other motor components and documenting their possible roles as mechanosensors are exciting areas of research moving forward.

## TWO DOCUMENTED MECHANOSENSORS IN BACTERIA

### FimH and catch and slip bonds: the first identified bacterial surface-sensing mechanosensor?

**FIG 5:**
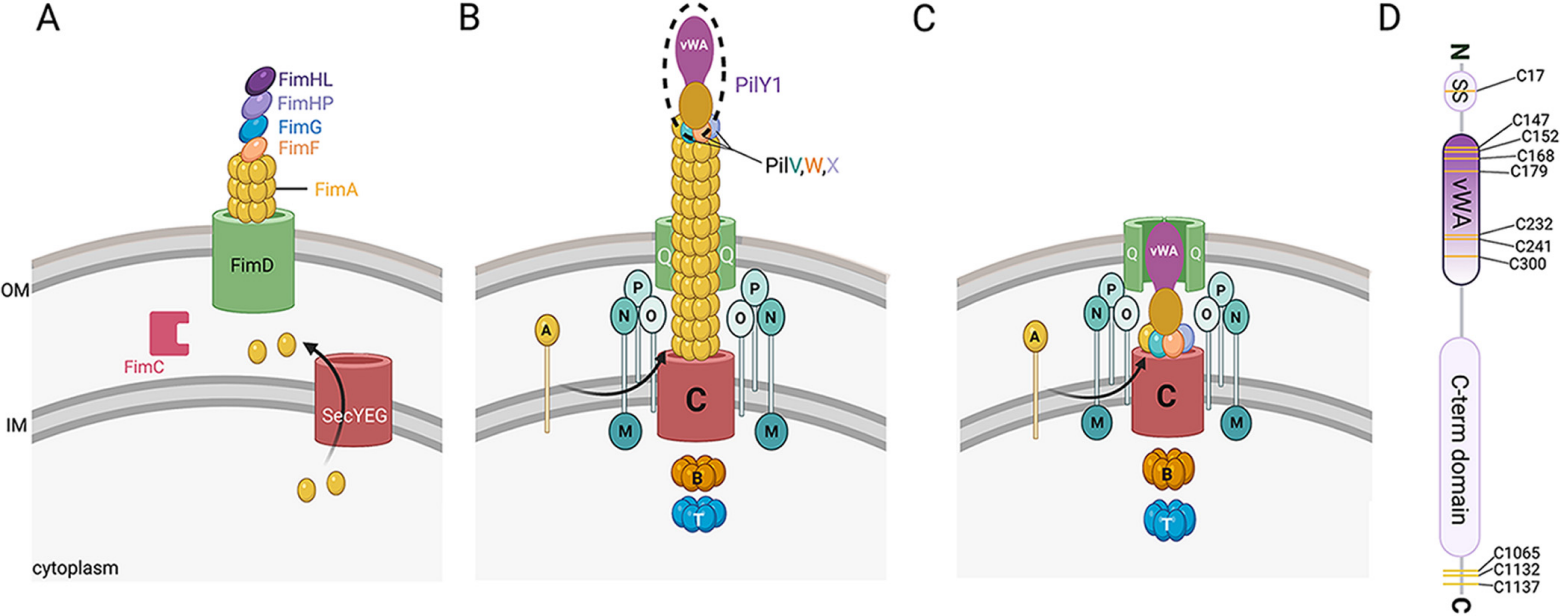
The E. coli type I fimbriae and P. aeruginosa type IV pili (TFP). (A) FimA subunits are translocated from the cytoplasm into the periplasm through the SecYEG system. The FimC chaperone binds and stabilizes the major fimbria subunit FimA, facilitating the FimA protein attaining its native conformation, wherein FimA interacts with the outer membrane protein FimD. FimA is subsequently incorporated into the fimbriae. The adhesin FimH, together with the minor pilins FimF and FimG, is located at the distal tip of the pili. FimF and FimG are likely important for the integration of FimH into the pili (162, 163). FimH is an ~30-kDa protein consisting of two domains, an N-terminal mannoside-lectin binding domain (FimHL) and a C-terminal pilin domain (FimHP). Unlike the TFP, the type I fimbriae do not retract and are much shorter. (B) The TFP machinery: secretin (PilQ [green]), platform protein (PilC [red]), motors (PilB and PilT [orange and blue, respectively]), the alignment complex (PilMNOP [shades of aqua]), and pilus fiber (PilA monomers [yellow]). During extension, PilA monomers in the inner membrane (IM) are incorporated into the growing pilus, pushing the priming complex to the tip of the extended pilus, with PilY1 at the apex. (C) The TFP machinery with the pilus fully retracted. (D) Schematic showing the domain organization of PilY1. The signal sequence (SS), vWA domain, and C-terminal domain are shown. Yellow stripes represent the cysteines present in the protein. Seven of the 11 total cysteine residues are located in the vWA domain, with the signal sequence and the C-terminal region having 1 and 3 cysteine residues, respectively. PilY1 is ~120 kDa, and the vWA domain is ~26 kDa.

Shear forces were initially shown to prevent bacterial attachment to surfaces due to the slip bonds that form between the bacteria and the substrate, which are weakened with increasing force (46, 105–107). However, catch bonds were subsequently shown to play a role in both Gram-positive and Gram-negative bacteria in the context of bacterial biofilm formation in the presence of flow (108, 109). For example, Thomas et al. demonstrated that the type I fimbrial adhesin protein FimH of E. coli (Fig. 5A) increased binding to target cells when shear forces were increased 10-fold (109), suggesting the presence of FimH-mediated catch bonds that are strengthened by increasing mechanical force (110). FimH is located at the tip of the type I fimbriae and has two domains that interact (111), a pilin domain (FimHP) that is incorporated into fimbriae and a lectin domain (FimHL) that binds the carbohydrate mannose, found on human cells (112). These domains are connected by a 3-amino-acid interdomain linker peptide (113). Mutations in either the pilin or lectin domains that disrupt their interaction substantially enhance the binding affinity of the lectin domain for mannose (111). A purified lectin domain or the addition of a chaperone molecule that is wedged in the interdomain region (thus disrupting the pilin-lectin domain interaction) also increases the binding affinity for mannose, supporting the finding that domain interaction inhibits mannose binding to the lectin domain.

The FimA monomers comprise most of the type I fimbriae of E. coli (similar to PilA for the P. aeruginosa TFP): at the tip are FimF and FimG (analogous to the minor pilins in P. aeruginosa), with FimH at the apex (analogous to PilY1 in P. aeruginosa) (Fig. 5B and C) (114). When force is applied, the fimbriae uncoil and are stretched, and FimH increases its binding to mannose (115, 116). A single type I fimbria that is pulled at a constant force using AFM, with a cantilever tip functionalized with mannose, shows a stepwise ~5-nm increase in the length of the fimbria that corresponds to the approximate length of the FimA and FimH subunits (117). Force-dependent binding to mannose could explain the observed correlation between shear forces and binding affinity for mannose; that is, under low forces, the binding affinity for mannose is low and vice versa (47). Analogous to vWF binding to GpIbα, FimH-mannose binding also displays a biphasic pattern reminiscent of the catch-slip bond regime, where an initial increase in the bond force and bond lifetime is observed, followed by a decrease. This catch-slip bond transition has also been observed between fibrinogen and the surface protein SpsD and clumping factor A (ClpA) in Staphylococcus aureus (108, 118).

Together, these studies indicate a mechanism where high shear forces cause conformational changes in FimH that dissociate the lectin from the pilin domain, which in turn causes the switch from low-affinity to high-affinity binding to mannose via a catch bond mechanism (111, 119). The FimH interdomain interaction decreases with increased force due to the transition to a slip bond regime, and with a subsequent reduction of the shear force, the protein transitions back to its original low-affinity globular conformation. Thus, we argue that FimH is likely the first identified mechanosensor with a documented role in surface engagement during the early stages of biofilm formation. What is lacking is strong evidence for a subsequent signal transduction pathway (e.g., mechanotransduction).

### TFP and the PilY1 mechanosensor in surface sensing.

The TFP machinery is well conserved across many species of bacteria, and consistent reports of its structure have emerged (112, 120–122). In Gram-negative organisms, the TFP machinery consists of more than 10 different proteins, which form the secretin, platform, motors, alignment complex, and pilus fiber (Fig. 5B and C). This molecular machine extends from the inner membrane (IM) to the outer membrane (OM) and beyond, potentially allowing the sensing and transduction of external surface signals into the cytoplasm. The PilQ multimer forms the secretin pore in the OM and serves as the channel through which the pilus fiber is spooled. PilC, located in the IM, is the platform protein and directly interacts with the ATPase motor proteins PilB and PilT/U, which control the elongation and retraction of the pilus, respectively. The PilMNOP alignment subcomplex links the secretin to the motor proteins and surrounds the pilus fiber, providing structural support during assembly. The alignment complex is also important in c-di-GMP signaling, highlighting the dual functionality of these proteins (123). The pilus fiber is made of the major pilin subunit PilA. PilA monomers assemble through interactions between their conserved N-terminal hydrophobic α-helices, which are buried in the core of the fiber (124). The priming complex consists of the minor pilins FimU and PilVWXE and the tip-associated protein PilY1 (125–127) (Fig. 5B and C). Together, the minor pilins and PilY1 facilitate the incorporation of PilA monomers into the base of the pilus fiber to initiate elongation. Eventually, the growing structure pushes the priming complex, including PilY1, to the very tip of the growing pilus. Cryo-electron tomography studies show that the vWA domain of PilY1 is situated at the extreme tip of the pilus (Fig. 5B and C), indicating that this domain can make direct contact with a surface and thus has the potential to be a mechanosensor (125).

TFP have long been associated with the formation of bacterial biofilms and surface-associated twitching motility (73, 128–130). TFP are characterized as one of the most powerful molecular machines, with a dynamic structure that functions through repeated rounds of extension, attachment, and retraction of the pilus, pulling the cell body forward in a jerky motion known as twitching motility. When the pilus adheres to a surface and is then retracted, a remarkably high force is generated (131, 132). This force is typically ~30 pN in the case of P. aeruginosa but can be as high as 250 pN (133). TFP can also generate forces in the nanonewton range, as seen for the bundling of the TFP in cooperative retraction, i.e., Neisseria gonorrhoeae (134).

### (i) How might the TFP cellular appendage sense surface engagement?

While the TFP is often stated to be a mechanosensor, evidence for this claim is largely lacking, is deductive, and/or relies on downstream phenotypic outputs. In current models, TFP are proposed to be responsive to mechanical forces experienced during surface engagement (132, 135, 136). Planktonic cells can readily extend and retract their pili. In proximity to a substratum, pili can bind the surface; in the latter scenario, the pilus cannot be readily retracted. For example, if one pilus is bound, retraction of the TFP would result in the cell body moving toward the surface. If two or more TFP are bound, they may be working in opposition. Given that the adhesive force of P. aeruginosa on a surface mediated by TFP averages ~150 pN (133), which is higher than the typical retraction force of the TFP (typically ~30 pN for a naive surface [137]), a tug-of-war may ensue when two or more TFP are bound and retracting. The resulting conformational changes in the pilus under force may include bond breaking, bond making, bond strengthening, bond weakening, or changes in the overall fiber structure (124, 138, 139), which likely occur when the pilus is stretched. Pilus stretching can also reveal epitopes buried in PilA (124). Below, we detail some of the changes that TFP may undergo when encountering a surface.

As noted above, some components of pilus-generated force can be in opposition and partly cancel. In contrast, there may also be components of force that are not canceled and so result in movement. These ideas are explored in Marathe et al.’s “tug-of-war” model and Jin et al.’s “slingshot” model (140, 141). The latter describes what happens when a pilus-to-surface attachment point ruptures during a tug-of-war between pili. It is clear that additional study will be required to understand how multiple TFP simultaneously interact with a surface to generate specific force profiles.

### (ii) Conformational changes in the TFP: plateaus and spikes.

When the TFP of P. aeruginosa attaches to a substratum, AFM studies have revealed that this fiber and its tip-associated adhesin PilY1 display two distinct adhesion force behaviors in response to surface-engagement-dependent mechanical tension, namely, plateaus and spikes (133). Plateaus in AFM analysis are defined as adhesive events with a “step” behavior, i.e., a constant sustained force over a defined length. Plateau signatures are explained by three possible mechanisms. First, there may be a progressive desorption of the pilus fiber from the surface; that is, the pilus is slowly released from the surface. However, since the dwell time (time between attachment and detachment) increases when there is tension on the pilus, this mechanism is thought to be less likely (142). Second, there may be adhesion of the pilus fiber at multiple points along the entire length of the pilus to the surface, not just the tip (138, 143), as in type I pili of Lactobacillus rhamnosus GG, which attach at multiple points along the length of the pilus when exposed to low shear forces. Distinct differences observed in the force plateaus of P. aeruginosa attached to a variety of hydrophobic and hydrophilic surfaces are also attributed to this zipper-like adhesion pattern (138). Third, force plateaus can result from conformational changes due to the elongation of the pilus fiber or TFP-associated proteins (124). Surface-attached P. aeruginosa display spike behaviors. Spikes are sharp events or force peaks with a single minimum and are typical of nanospring-like behaviors (133, 144). This behavior suggests that TFP have elastic properties such that after stretching, once force is no longer applied, the pilus rapidly returns to its original shape. Pili of L. rhamnosus GG also show these nanospring properties (143); the protein SpaC, the key adhesion protein of the L. rhamnosus GG pilus, behaves like a nanospring at high forces. Compared to the P. aeruginosa TFP, the nanosprings of L. rhamnosus GG pili are able to sustain much higher forces as the pili of this Gram-positive organism are formed by covalent polymerization. It is likely that this nanospring behavior allows this bacterium to sustain higher forces when the fiber is stretched, allowing the pilus to engage the surface with maximum force without snapping.

These features of TFP underscore that the pilus is capable of maintaining both integrity and elasticity under force. The findings described above (particularly the observations regarding how plateau signatures are generated) raise the important question of whether the TFP of P. aeruginosa are attaching at the tip or over the pilus’s entire length, a point discussed in more detail below.

### (iii) Current models for TFP-mediated mechanosensing and signal transduction.

How might a mechanical signal from surface contact that is propagated by the TFP be translated into a biochemical response in the cytoplasm? One model proposes that the TFP acts as a force sensor by detecting resistance to retraction, which in turn generates a signal that is relayed intracellularly and results in increased second messenger production (136, 145). For example, Ellison et al. found that physically blocking pilus retraction in Caulobacter crescentus using polyethylene glycol 5000 (PEG 5000)-maleimide was sufficient to stimulate holdfast production (136). Those authors conclude that the obstruction of TFP retraction during surface contact serves as a mechanical input that activates a signaling pathway modulating c-di-GMP levels and stimulating holdfast synthesis (136). However, how this signal is transmitted from the TFP into the cell is unclear. Alternatively, might the addition of PEG 5000-maleimide simply impede flagellar rotation, allowing us to invoke the model proposed by Hug et al. (100)? Or are apparently conflicting findings on the respective roles of TFP and flagellar motors in signaling the result of the different experimental setups used? Additional study will be required to sort out this conundrum.

In another model that implicates the TFP as a force sensor, Persat and colleagues suggest that when the TFP is bound to a surface and is under tension, PilA adopts a new stretched conformation that interacts with PilJ; this interaction, in turn, is proposed to activate the Pil-Chp system, which ultimately results in increased intracellular cAMP levels (145). A contemporary study by Luo et al. also implicates the Pil-Chp-cAMP response in surface sensing (75) but does not invoke a mechanosensing mechanism. The model posited by Persat et al. raises two possibilities for signal transduction via the PilA-PilJ interaction. First, the PilA-PilJ interaction occurs when PilA is still incorporated into the pilus fiber. Second, the interaction occurs when PilA is in the IM after being removed from the pilus fiber during retraction. In the first instance, it is likely that PilC, together with the priming complex and PilY1, sterically blocks the interaction between PilA and PilJ. Thus, a PilA-PilJ interaction while PilA is still incorporated into the pilus machine would be unlikely. Is it possible that PilA maintains the stretched conformation when in the IM after being released from the pilus in the absence of tension? If so, how would PilA maintain an altered conformation in the absence of tension? To test the latter model, perhaps one could determine whether it is possible to isolate mutations that lock PilA in this signaling conformational state, thus showing constitutive downstream cAMP signaling. If this model is correct, and signaling via PilJ is indeed required for surface sensing, presumably, such PilA mutants would show an interaction with PilJ or, at the very least, require PilJ for cAMP signaling.

Koch et al. recently proposed that local concentrations of PilA in the IM change during TFP extension and retraction and in a substratum-dependent manner (135). Those authors suggest that these rapid temporal changes in the PilA concentration are sensed by the PilJ-Chp chemosensory system, wherein PilA likely interacts with PilJ. However, the question remains as to whether the PilA monomers that diffuse into the IM as the pilus fiber is retracted are able to retain force-induced conformational changes or if it is simply a case of the local PilA concentration driving signaling. That is, does PilJ interact with PilA only when there is a locally high concentration in the IM during retraction, a model at odds with most of the currently published data (75, 145–147)? If the latter scenario is true, invoking a model in which the TFP are a mechanosensor by this group was premature. Furthermore, we must also consider that the levels of PilA are regulated in part by their abundance in the IM. Kilmury and Burrows (148) proposed an “inventory control” model whereby the abundance of PilA in the IM autoregulates the transcriptional control of *pilA* expression, which would impact any model, including that of Koch and colleagues (135), that invokes monitoring PilA levels.

### (iv) Mechanical forces and the cysteine residues of the vWA domain of PilY1: evidence for a bacterial mechanosensor.

PilY1 is a 120-kDa protein that has a C-terminal alcohol dehydrogenase-like domain (149) and an N-terminal vWA domain structurally similar to the A2 domain of the human vWF (Fig. 3C). vWA-containing proteins are involved in a variety of functions and are found in all domains of life (150). The vWA domain, including that of PilY1, has the classical Rossman fold—central β-sheets surrounded by amphipathic α-helices (151)—and a perfectly conserved metal ion-dependent adhesion site containing the DxSxS motif and the noncontiguous T and D residues (152). Relative to the rest of the protein, the vWA domain has a high abundance of cysteine residues. Seven of the 11 cysteines in PilY1 are in the vWA domain (Fig. 5D), and recent genomic studies showed that all 7 cysteines are conserved in 99% of strains of the P. aeruginosa PA14 clade analyzed from the International Pseudomonas Consortium Database, while only 5 of the 7 cysteines are conserved in 99% of a total of 723 P. aeruginosa PAO1 lineage strains (144).

The hypothesis that mechanical forces can drive mechanosensing by PilY1 is plausible based on our genetic analysis, discussed elsewhere (75, 97), and the overview presented above for the mechanosensitive proteins titin and vWF. Here, we make inferences about PilY1 function from the studies of titin and vWF and present a possible mechanism for force-induced mechanosensing utilizing the vWA domain of PilY1.

The A2 domain of vWF and the I27 domain of titin undergo folding/unfolding events in response to force (22, 37). These domains adopt a stretched or elongated conformation when force is applied and transition to a globular conformation when the force is removed; thus, these proteins satisfy the classical definition of a mechanosensor by undergoing force-dependent (and, in these cases, reversible) conformational changes (14). During mechanosensing, the pilus, and in particular the vWA domain of PilY1, is likely to be in direct contact with the surface (as discussed above), and PilY1 is therefore likely subjected to mechanical force. Based on findings for titin and vWF, we postulate that when the surface-bound pilus begins to retract, the mechanical forces due to surface contact not only cause the PilA monomers in the pilus fiber to change conformation (analogous to what is observed for N. gonorrhoeae TFP under force [124]) but also cause the vWA domain of PilY1, the TFP tip-associated adhesin (153), to adopt a partially unfolded and/or stretched conformation. It is important to note that the change in conformation proposed here is distinct from the “melted” domain of PilA that has been observed as a characteristic of TFP of P. aeruginosa and *Neisseria* spp. (124, 154). Our AFM studies show that when the force is removed, TFP/PilY1 reverts to a folded state, highlighting the flexibility and the dynamic nature of the TFP and/or the vWA domain of PilY1 (144). It is important to note that it is not clear if the refolded state after the application of force is identical to the protein’s original conformation. That is, an additional possible layer of impact on TFP/PilY1 is that force induces a new, novel conformation that could impact signaling, an idea worth further investigation.

Cysteines in vWF and titin play a major role in force-induced conformational changes wherein the redox states of disulfide bonds in the A2 domain of vWF and the I27 domain mediate unfolding and refolding events (22, 37, 41, 44). Given the high number of cysteines in the vWA domain of PilY1, one or more disulfide bonds may act as a redox switch that drives unfolding and refolding events or may stabilize the PilY1 protein as the cell encounters forces due to surface contact. Although the vWA domain of PilY1 does not have a vicinal sulfhydryl, it does have two cysteines, Cys147 and Cys152, that are important in biofilm formation and c-di-GMP signaling (144).

Recalling the above discussion of spikes and plateaus, AFM studies have shown that WT PilY1 displays an approximately equal number of these features, while mutating the Cys152 of PilY1 alters the ratio of plateaus to spikes from ~50:50 to ~10:90 for surface-engaged cells, with a concomitant loss of the surface-dependent induction of c-di-GMP production. No such change in protein conformation is noted for the wild-type versus the Cys152Ser mutant PilY1-vWA domain in solution (144). Furthermore, the Cys152Ser mutation does not impact twitching motility, effectively separating the effects of PilY1 on TFP assembly/function from surface sensing. Could these cysteines be involved in unfolding and refolding events during surface sensing? If so, this suggests that mechanical forces encountered by the pilus and PilY1 during surface sensing could produce enough force for the protein to adopt a stretched or elongated conformation. Alternatively, disulfide bonds could be broken by the force generated by the retraction of surface-bound TFP. While the maximum retraction or binding forces generated by P. aeruginosa (<250 pN) are below the nanonewton forces typically required to break disulfide bonds (63), it is formally possible that TFP attachment could alter the bond angles and degrees of freedom, allowing much lower forces to break a disulfide bond. The forces needed to break disulfide bonds have been reported to be as low as 100 pN (62), which fall within the range of forces that these microbes experience. Therefore, tuning the protein redox state of disulfide bonds using mechanical forces is a mechanism that microbes are likely to use to detect surface contact.

As is the case for the A2 domain of vWF, it is likely that spontaneous refolding of the vWA domain takes place in the absence of force, a conclusion consistent with AFM studies (144). We propose that whether the protein attains a new, unstable, force-dependent conformation or the disulfide bonds of the vWA domain of PilY1 are broken during surface contact, the surface-engaged PilY1 protein would be in a high-energy state, and therefore, the reverse reaction, that is, protein folding, is likely spontaneous when the mechanical force is removed (Fig. 1). Thus, we predict that during retraction events when the pilus is no longer in contact with the surface and, thus, not subject to mechanical force, the vWA domain spontaneously refolds to its native conformation. This spontaneous reformation, mediated by the disulfide bond(s) in PilY1, would be critical for resetting the system. Together, these analyses of PilY1 provide key data linking force-dependent conformational changes to changes in cellular signaling in a bacterial system.

Finally, to build out any model, we must address the question of whether TFP attach by the tip or over their entire length. If adhesion occurs along the length of the pilus fiber (composed of PilA), we expect the occurrence of force plateaus between WT PilY1 and the Cys152Ser mutant to be the same. Second, force plateaus can result from conformational changes due to the elongation of the pilus fiber as it experiences mechanical tension from being pulled at the tip. For example, in N. gonorrhoeae, a new conformation that is three times longer and 40% narrower than the unstretched or original structure is observed when the pilus is pulled at the tip with a force of 100 pN, revealing the EYYLN epitope of the PilA subunit that is buried within the pilus fiber (124). For WT P. aeruginosa, the pilus is also observed to be straight when under force, distinct from the floppy pilus observed for mutant strains lacking the PilT retraction motor (142). Taken together, and consistent with another recent report (135), these results show that TFP likely bind to the surface via their tip-associated adhesin PilY1.

In total, the observations outlined above are consistent with a proposed mechanism in which PilY1 at the TFP tip attaches to the surface, and PilY1 experiences a force-induced conformational change (i.e., the observed plateaus and spikes in AFM studies) upon the retraction of adhered TFP. The force generated by TFP retraction drives the sustained conformational change of the vWA domain of PilY1, which can be maintained as the pilus fully retracts. We speculate that if the PilY1 tip adhesin of the fully retracted pilus is still surface bound, binding would likely occur via the vWA domain of PilY1, which is located at the very tip of the pilus. Based on cryo-electron microscopy (cryo-EM) and pulldown studies in *Myxococcus* (125), fully retracted PilY1 can interact with PilO (Fig. 5C), a component of the TFP alignment complex and an outside-in c-di-GMP signaling system (123). Thus, we posit that PilY1 in the resulting mechanically induced conformation would be able to interact with PilO. Based on the published cryo-EM structure of the *Myxococcus* TFP (125) and our previous observation that the C terminus of PilY1 is dispensable for signaling (155), we predict that the vWA domain of PilY1 would interact with PilO. In this model, this PilY1-PilO interaction sequesters PilO from SadC, an inner membrane diguanylate cyclase, leading to SadC activation and increased c-di-GMP levels (123) and thereby promoting biofilm formation.

Based on the evidence described above, PilY1 satisfies the classical definition of a mechanosensor (14). However, the discussion above also raises several important questions given that TFP play so many roles. TFP are surface-sensing organs, motility appendages, and adhesins; it is unlikely that each of these roles requires the same type of mechanical response to different magnitudes of force. In the original conception, catch bonds allow leukocytes to “roll” under high-shear conditions without getting stuck under low-shear conditions. Can something similar happen for TFP? For example, can the structure of PilY1 allow strong adhesion at high forces but also allow sensing at lower forces? To generalize the question further, does the vWA domain increase the bond strength and interaction with the surface as the force on the TFP increases, and does an increase in PilY1-mediated bond strength decrease once some critical force is reached? Is it possible for the system to display catch bond/slip bond behavior? If there were an analogy to catch bonds, how is this implemented in this specific physiochemical system? Could the formation of catch bonds, and a subsequent transition to slip bonds, alter the conformation of the vWA domain, and could this transition drive signaling? Given that force is vectorial in nature, does this imply that TFP force applied to PilY1 in different directions may have different conformational outcomes? Also, is there a chemical component to mechanosensing? Due to the polarizability of divalent sulfur, the S-S bond can be significantly weakened by the presence of either Lewis bases or Lewis acids; do the oxidizing conditions of the periplasm “reset” disulfide bonds in the system? These are all open questions worthy of future investigation.

## CONCLUSIONS AND OUTLOOK

Bacterial mechanobiology is an emerging and exciting field. During the past decade, progress has been made in understanding the mechanisms of surface sensing. The preponderance of data at this time supports the idea that bacteria can sense surface engagement. In contrast, deploying words like “mechanosensory” and “mechanosensing” in the context of bacteria sensing a surface is often premature. An exciting frontier is understanding how physical signals are sensed and transmitted by bacteria and how such physical inputs are converted to biochemical signals.

Currently, it is unclear how many examples of surface sensing have a direct role for mechanosensing proteins like PilY1 and FimH. That is, in the case of the stators sensing a surface or TFP stimulating cAMP production upon surface contact, is there a direct mechanical signal involved, or is the signal indirect (i.e., the loss of flagellar rotation alters stator occupancy, or the concentration of the PilA monomer in the inner membrane changes)?

Disulfide bonds serve as a powerful framework to think about questions of surface sensing, but this is unlikely to be the only framework. In fact, it is almost certain that multiple mechanisms are at play given the different ways in which bacteria attach to surfaces. That is, there are clear distinctions in the mechanisms of early biofilm formation among motile and nonmotile organisms, between Gram-positive and -negative organisms, and even among the pseudomonads (156–158). Nonetheless, what has become clear is that there is a great need for interdisciplinary research teaming microbiologists and biophysicists to truly understand mechanosensing.
